# Comparing effects of Euclidean buffers and network buffers on associations between built environment and transport walking: the Multi-Ethnic Study of Atherosclerosis

**DOI:** 10.1186/s12942-022-00310-7

**Published:** 2022-09-17

**Authors:** Jingjing Li, Adam Peterson, Amy H. Auchincloss, Jana A. Hirsch, Daniel A. Rodriguez, Steven J. Melly, Kari A. Moore, Ana V. Diez-Roux, Brisa N. Sánchez

**Affiliations:** 1 Department of Land Resources Management, School of Public Administration, China University of Geosciences, 388 Lumo Rd., Hubei 430074 Wuhan, China; 2 Urban Health Collaborative, Dornsife School of Public Health, Drexel University, 3600 Market St. 7th Floor, PA 19104 Philadelphia, USA; 3 Department of Biostatistics, the University of Michigan, 1415 Washington Heights, Ann Arbor, MI 48109 USA; 4 Department of Epidemiology and Biostatistics, Dornsife School of Public Health, Drexel University, 3215 Market St, Nesbitt HallPhiladelphia, PA 19104 USA; 5 Department of City & Regional Planning and Institute for Transportation Studies, University of California Berkeley, 230 Wurster Hall #1820, Berkeley, CA 94720 USA

**Keywords:** Built environment, Transport walking, Simulation, Network buffers, Euclidean buffers

## Abstract

**Background:**

Transport walking has drawn growing interest due to its potential to increase levels of physical activities and reduce reliance on vehicles. While existing studies have compared built environment-health associations between Euclidean buffers and network buffers, no studies have systematically quantified the extent of bias in health effect estimates when exposures are measured in different buffers. Further, prior studies have done the comparisons focusing on only one or two geographic regions, limiting generalizability and restricting ability to test whether direction or magnitude of bias are different by context. This study aimed to quantify the degree of bias in associations between built environment exposures and transport walking when exposures were operationalized using Euclidean buffers rather than network buffers in diverse contexts.

**Methods:**

We performed a simulations study to systematically evaluate the degree of bias in associations between built environment exposures in Euclidean buffers and network buffers and transport walking, assuming network buffers more accurately captured true exposures. Additionally, we used empirical data from a multi-ethnic, multi-site cohort to compare associations between built environment amenities and walking for transport where built environment exposures were derived using Euclidean buffers versus network buffers.

**Results:**

Simulation results found that the bias induced by using Euclidean buffer models was consistently negative across the six study sites (ranging from -80% to -20%), suggesting built environment exposures measured using Euclidean buffers underestimate health effects on transport walking. Percent bias was uniformly smaller for the larger 5 km scale than the 1 km and 0.25 km spatial scales, independent of site or built environment categories. Empirical findings aligned with the simulation results: built environment-health associations were stronger for built environment exposures operationalized using network buffers than using Euclidean buffers.

**Conclusion:**

This study is the first to quantify the extent of bias in the magnitude of the associations between built environment exposures and transport walking when the former are measured in Euclidean buffers vs. network buffers, informing future research to carefully conceptualize appropriate distance-based buffer metrics in order to better approximate real geographic contexts. It also helps contextualize existing research in the field that used Euclidean buffers when that were the only option. Further, this study provides an example of the uncertain geographic context problem.

**Supplementary Information:**

The online version contains supplementary material available at 10.1186/s12942-022-00310-7.

## Introduction

Transport walking (walking for transport purposes) has drawn growing interest due to its potential to increase levels of physical activity and reduce reliance on motor vehicles. Researchers have investigated the impact of place-based built environment features on transport walking [27]. Higher residential density [7], higher accessibility to amenities [19], well-connected street networks [15, 30], and higher land use mixture [16] may encourage higher levels of walking for transport. However, area-based measures of built environment exposures are susceptible to the uncertain geographic context problem [17] (i.e., the deviation of a defined spatial unit from the geographic context that is most relevant to an individual’s experience) and this problem may influence empirical estimates of the effects of built environment on health behaviors of interest. Thus, it is essential to examine how different definitions and delineations of spatial contexts influence the assessment of built environment exposures [26], which will help identify the spatial context that best approximates the actual geographic context an individual experience [18, 28].

There are two common ways to delineate a spatial context within which built environment features are measured: Euclidean buffers and road network buffers [14]. Euclidean (also called radial or as-the-crow-flies) buffers are created by drawing a straight line from a location and using that line as the radius of a circle [2], whereas road network buffers are irregular shapes created by drawing line segments from a location along road networks and including a specified distance along the network from the location [9]. We will use the term “network buffer” to represent “road network buffer” for simplicity. Euclidean buffers are easier to compute, especially for areas that lack digital information on road networks,however, they may not accurately reflect spatial access via automobile, bicycle, foot or other travel modes [22]. By comparison, network buffers account for travel routes along road networks, which may be preferred by those aiming to quantify walking or biking access to an amenity using the road system [5]. Network buffers require high quality road data which may not always be available. Additionally, calculations of network buffers are computationally intensive, particularly for large study areas and/or a large number of subjects or built environment features of interest.

Given the advantages and disadvantages of Euclidean buffers and network buffers, it is important to quantify differences in built environment exposures derived from them, and investigate the direction and extent of bias in the subsequent estimates of built environment-health associations. Specifically, it is important to determine under what conditions the results from the two methods are roughly equivalent and under what conditions the two methods are highly divergent, and explore whether the magnitude of the differences varies across geographic contexts. Examining the differences in health impacts of built environment exposures measured with Euclidean buffers and network buffers will help clarify when the more computationally intensive network-based calculations are justified, and also help inform findings from past (or even future) studies where Euclidean buffers are the only option. This study employs simulation modeling to systematically quantify how built environment exposures measured in Euclidean buffers and network buffers influence transport walking.

Previous studies have empirically examined variations in health impacts of built environment exposures measured in buffers with varying shapes and sizes [4, 6, 10] or in administrative units [21]. For instance, some studies compared effects of Euclidean and network buffers on associations between built environment and health behaviors [14, 22, 29]. They found that the associations between built environment and health behaviors were stronger for those measured in network buffers. While existing studies have compared built environment-health associations between Euclidean buffers and network buffers, no studies have systematically quantified the extent of bias in health effect estimates when built environment exposures were measured in Euclidean buffers versus network buffers. Further, prior studies have done the comparisons focusing on only one or two geographic regions, limiting generalizability and restricting ability to test whether direction or magnitude of bias are different by context. Consequently, there is a need to obtain more generalizable knowledge about the extent of bias in health effect estimates when comparing these two types of spatial contexts (Euclidean buffers and network buffers).

This study aimed to investigate differences in the estimated associations between built environment exposures and transport walking when exposures were measured with Euclidean buffers or with network buffers. Our investigation first used a simulation study to examine this question. The advantages of simulations are that the researcher has control over the model inputs and thus can systematically test a wide variety of spatial scales, contexts, effect sizes, and density of built environment features. Thus, results will not be tied to a single dataset or area and can therefore be generalized to many contexts. Further, simulations allowed us to systematically evaluate the degree of bias when exposures were operationalized using Euclidean buffers rather than network buffers (assuming network buffers more accurately captured true exposures). Next, to anchor findings in empirical analyses, we examined the similar questions using empirical data from a multi-ethnic, multi-site cohort study. To enhance generalizability of study findings, we used two built environment exposure categories. The first category included destinations that were common in every-day life (a broad category of walkable destinations). The second category was a smaller subset of the first: frequent social destinations that facilitate social interaction and promote social engagement (e.g., beauty shop/barber). Prior work already confirmed their associations with transport working [19], and the differences in counts and densities between the two exposures allowed us to examine the way the prevalence of a feature impacts the bias.

## Data and methods

Below, we first describe the empirical dataset and the empirical analysis. Next, we describe how the empirical dataset was used to derive the simulated dataset and we described the simulation analysis.

### Empirical study

#### Multi-Ethnic Study of Atherosclerosis

Transport walking data and personal sociodemographic information came from Multi-Ethnic Study of Atherosclerosis (MESA), of which 6814 adults aged 45–84 years participated in the survey between July 2000 and August 2002 in six study sites in the U.S. (Los Angeles, California; Chicago, Illinois; Saint Paul, Minnesota; New York City, New York; Baltimore, Maryland; Forsyth County, North Carolina) [3]. MESA was approved by the institutional review boards at all participating institutions, and all participants gave written informed consent. Although MESA is a longitudinal study, we focused on the data at Exam 1 (between July 2000 and August 2002) because we were interested in investigating differences in estimated associations between built environment exposures and transport walking when exposure metrics were based on Euclidean buffers or network buffers—i.e., the longitudinal aspect of MESA did not add additional information on the question of interest in this study.

#### Outcome: transport walking minutes per week

The survey asked participants whether they had engaged in walking activities (walking to get to places e.g., to the bus, car, work, to stores) during a typical week in the past month. If yes, they were asked to report how many days per week and how much time per day they spent walking. The outcome variable transport walking minutes per week was computed by multiplying the number of days for transport walking per week by the number of minutes for transport walking per day. We log-transformed the outcome variable because it was skewed to the right.

#### Exposure: built environment

Built environment data came from the National Establishment Time Series (NETS) database, obtained through the MESA Neighborhoods study and Retail Environments for Cardiovascular Disease (RECVD) project (https://sites.google.com/view/recvd-team-project-site/home). Population density came from the 2000 U.S. Census (https://www.census.gov/). Road network data were obtained from ESRI Business Analyst 2016.

Built environment features included a broad category of walkable destinations, consisting of common and popular destinations for daily life (e.g., food stores, restaurants, drug stores and pharmacies, department stores, post offices, banks/credit unions, libraries, beauty shops and barbers, social/entertainment destinations, museums, schools). We further included one subset of the broad category of the walkable destinations: frequent social destinations to examine the variations in density for different built environment destinations. Frequent social destinations consisted of destinations that facilitated social interaction and promoted social engagement (e.g., beauty shop/barber, libraries, non-physical activity recreation clubs, religion). The detailed list of all the destinations is shown in Additional file 1: Table S1 (ST1). The exposure was defined as the number of destinations within the corresponding spatial context (either network or Euclidean buffer).

**Table 1:** Descriptive statistics for the MESA sample

	All (N = 5756)	NC (N = 891)	NY (N = 921)	MD (N = 864)	MN (N = 945)	IL (N = 995)	CA (N = 1140)
	Median (Q1-Q3)	Median (Q1-Q3)	Median (Q1-Q3)	Median (Q1-Q3)	Median (Q1-Q3)	Median (Q1-Q3)	Median (Q1-Q3)
Total transport walking minutes/week (minute)	150 (45–420)	150 (45–420)	225 (105–525)	150 (5–420)	120 (25–315)	210 (90–420)	105 (30–210)

	%	%	%	%	%	%	%
Female sex	52.3	52.6	56.9	51.7	50.2	53.0	50.1
Race/ethnicity							
White	39.2	54.8	21.0	50.1	58.5	47.2	10.6
Black/African American	26.9	45.0	35.2	49.9	0.0	26.6	11.1
Chinese	12.1	0.0	0.2	0.0	0.0	26.1	38.0
Hispanic	21.8	0.2	43.6	0.0	41.5	0.0	40.3
Education level							
High school/GED or less	34.7	28.5	42.7	29.2	39.4	14.8	51.0
Some college	28.3	29.6	28.1	29.7	35.0	23.9	24.6
BA or above	37.0	41.9	29.2	41.1	25.6	61.3	24.5
Currently employed	54.8	53.1	54.9	53.5	63.3	62.1	43.5
Own at least one car	83.0	98.1	45.7	91.1	92.6	85.5	85.0
Self-rated health compared with others							
Better	60.3	72.1	61.0	58.7	57.1	61.2	53.4
Same	34.8	24.6	34.0	37.0	38.4	34.7	38.9
Worse	4.9	3.4	6.0	4.3	4.4	4.1	7.7
Arthritis flare-up in past 2 weeks	12.3	12.3	11.4	15.7	9.6	12.8	12.1

	Mean (STD)	Mean (STD)	Mean (STD)	Mean (STD)	Mean (STD)	Mean (STD)	Mean (STD)
Age	61.83 (10.14)	62.3 (9.78)	61.5 (10.0)	63.0 (9.97)	60.0 (10.3)	62.0 (9.93)	62.4 (10.5)
Income and wealth index	4.74 (2.32)	5.78 (1.74)	3.50 (2.23)	5.42 (1.93)	4.83 (2.07)	5.83 (2.00)	3.42 (2.37)
Body mass index (kg/m^2^)	28.33 (5.40)	28.9 (5.29)	28.9 (5.54)	29.4 (5.49)	29.5 (5.27)	26.7 (5.03)	27.1 (5.12)
Number of population/mi^2^	15,380 (19,060)	1590 (857)	55,300 (13,800)	6860 (4460)	4720 (1600)	13,800 (6010)	10,600 (4630)
Street network ratio	0.42 (0.15)	0.23 (0.12)	0.49 (0.09)	0.39 (0.16)	0.45 (0.11)	0.47 (0.10)	0.47 (0.12)

Income and wealth index was created by adding together inflation adjusted per capita income (5 levels, ranges from 0 to 4) and wealth index (sum of home ownership, car ownership, land ownership, and investments, ranges from 0 to 4) [12]. *NC* North Carolina, *NY* New York, *MD* Maryland, *MN* Minnesota, *IL* Illinois, *CA* California

#### Covariates

Covariates were selected based on prior MESA studies which examined associations between transport walking and built environment features [13, 19]. Person-level covariates included age, sex, race/ethnicity, and education, income-wealth index, employment status, household car ownership, body mass index (BMI), self-rated health compared with others of the same age, and arthritis flare-up in the past 2 weeks. The income-wealth index was specified as a 9-point scale (0 being the lowest level of income and no assets and 8 being the highest level of income and all 4 assets). Details about the index are shown in the note of Table 1 and the index was described previously in depth [12]. Area-level covariates included population density in a 1-mile Euclidean buffer, street network ratio in a 1-mile Euclidean buffer, and region (from census categories: Northeast, Midwest, South, West). To calculate population density in a 1-mile Euclidean buffer around residence, first we used the 'intersect' geoprocessing tool in ArcGIS [GIS software] (Version 10.5. Redlands, CA: Environmental Systems Research Institute, Inc., 2016) to estimate the area weighted population for block groups/pieces of block groups within a 1-mile Euclidean buffer of each participant and then we divided the total population by the area of the buffer. Street network ratio in a 1-mile buffer was calculated as the ratio of the area of a 1-mile network buffer to the area of a 1-mile Euclidean buffer around each participant’s residence. The ratio varies between 0 and 1, with 0 meaning none of the area can be reached through the road network and 1 meaning the entire area can be reached through the street network (i.e., the highest level of network ratio).

#### Sample inclusion

There were 6191 MESA participants who agreed to participate in the MESA Neighborhood Study at Exam 1. We retained 5839 participants who had historical addresses and had geocoded addresses with accuracy at street and ZIP + 4 level. We excluded 15 participants who did not report transport walking minutes. We also removed 68 participants who did not have complete sociodemographic variables. The final analytical sample consisted of 5756 participants.

### Simulation study

#### Overview

Simulations were used to provide systematic evidence regarding how the two buffer methods (true exposure in network buffers and exposure in Euclidean buffers) perform on average. Because researchers have full control of how the data are simulated, the correct/true answer is known by design [1]. Thus, our simulations assessed which of the two methods came closest to recovering the ‘correct’ answer.

In order to add realism to the simulated dataset, simulations were based on the locations of MESA study participants and the spatial locations of amenities near participants. The outcome data transport walking minutes per week were generated according to a linear regression model (described below). Note that to generate the transport walking minutes per week, we used the observed built environment exposures in network buffers as the true exposure because buffers delineated through street network may be assumed to be a more precise representation of access by walking or other active travel [22].

#### Simulation design

We simulated transport walking minutes per week under a variety of settings in which the outcome was dependent upon spatial accessibility and a binary covariate. Simulations were designed to examine bias in built environment-outcome association estimates resulting from using Euclidean buffer counts as the observed predictor (denoted ${X}^{Euc}$), but outcomes were generated using network buffer counts as the true predictor (denoted ${X}^{Net}$). That is, for a subject *i*, we generated data from a model: ${Y}_{i}=\alpha +{\varvec{\gamma}}{{\varvec{Z}}}_{{\varvec{i}}}+\beta {X}_{i}^{Net}+{\varepsilon }_{i}$, and sought to determine the degree of bias in estimates of the $\beta$ coefficient when ${X}_{i}^{Euc}$ is used instead of ${X}_{i}^{Net}$. To obtain generalized understanding of patterns in the degree of bias, we used 72 simulation settings, which arose from the combinations of: three spatial scales (0.25 km, 1 km, 5 km), two types of built environment features (BEF), two effect sizes (smaller/larger), and six geographic contexts (the six MESA sites).

We chose 0.25 km, 1 km, and 5 km to represent a small, a medium, and a large spatial scale, respectively. These distances were selected because they align with prior work in this field and/or can be justified. We employed a spatial–temporal aggregated predictor (STAP) modeling [24] to detect how associations between walkable destinations and transport walking varied across distances, and found that associations were negligible for distances larger than 0.25 km, which was in line with findings in a previous MESA study that smaller spatial scale of 0.2 km had stronger effects than larger ones [25]. Prior work among adults has widely used 1 km (equivalent to a 10–15 min walking distance) to represent the size of a residential neighborhood [8, 11, 20, 23], and 5 km (~ 3.11 mile) represents the maximum distance because most US residents are unwilling to walk for transport farther that this [31].

We examined two classes of overlapping BEFs: Walking Destinations (WD) and Frequent Social Destinations (FSD, a subset of the former) to ensure differences in results from a dense (WD) vs. less dense (FSD) BEF were not due to differences in the spatial distribution of the features. We used a smaller (0.05) and larger (0.1) built environment effect, $\beta$, to examine if biases depended on the magnitude of the effect size. For each of the 72 simulation settings, we simulated 5000 datasets.

#### Analysis of simulated data

For each simulated dataset, we employed the count of built environment destinations in the network or Euclidean buffers as predictors in separate models, and estimated their association with the generated outcome. For example, to estimate the association between WD in 1 km network buffers and outcome, we fitted $E\left[{Y}_{i}\right]=\alpha +{\varvec{\gamma}}{{\varvec{Z}}}_{{\varvec{i}}}+{\beta }_{Net\_1km}{X}_{WD,i}^{Net\_1km}$. Similarly, we fitted a similar model using the WD counts within 1 km Euclidean buffers $E\left[{Y}_{i}\right]=\alpha +{\varvec{\gamma}}{{\varvec{Z}}}_{{\varvec{i}}}+{\beta }_{Euc\_1km}{X}_{WD,i}^{Euc\_1km}$. We evaluated the performance of the two buffer metrics in estimating associations by comparing estimates to the true value and averaging across simulations $\frac{1}{5000}{\sum }_{s=1}^{5000}({\widehat{\beta }}_{s,Net}-\beta )$, as well as comparing the differences in associations, namely $\frac{1}{5000}{\sum }_{s=1}^{5000}({\widehat{\beta }}_{s,Euc}-{\widehat{\beta }}_{s,Net})$.

We obtained an estimate of the bias in the coefficient estimates by comparing the estimate obtained in each dataset to the true value and averaging across the 5000 simulated datasets for a given scenario. We standardized the bias into a percent, by dividing the average bias by the true coefficient, and plotted the percent bias to visualize the results. For each simulation scenario, we also obtained an estimate of power to detect significant associations by calculating the percent out of the 5000 datasets where the confidence interval for β did not contain zero.

### Empirical analysis of MESA data

We calculated descriptive statistics for all MESA study variables. We focused on quantifying the extent of differences in the built environment exposures when they were measured using Euclidean or Network buffers with varying buffer sizes (0.25 km, 1 km, and 5 km) among MESA participants. Differences in exposure metrics were summarized as median, the first quartile, and the third quartile. We estimated associations between transport walking and built environment exposures assessed using network buffers and Euclidean buffers. Although we used three spatial scales for the buffers: 0.25-km, 1-km, and 5-km, we ultimately focused on 0.25-km buffer models as the health effects of walkable destinations beyond 0.25-km (in network distance) were weak.

## Results

### Descriptive statistics for the analytical sample

Table 1 shows the descriptive statistics for the analytical sample. The sample was 52% female, 39% white, 37% having bachelor’s degree or above, and 55% were employed. Mean participants’ age was 62 years (SD: 10.14). About 83% of participants owned at least one car. Participants’ mean BMI was approximately 28.3 kg/m^2^ (SD: 5.40 kg/m^2^). The median number of minutes of self-reported weekly transport walking was 150 (Q1-Q3: 45–420). The mean population density of participants’ 1-mile neighborhood was 15,380 (SD: 19,060). Mean street network ratio was 0.42 (SD: 0.15). Meanwhile, the sample showed diverse characteristics across the six sites. Participants from New York City and Chicago walked more for transportation with median values of 225 min and 210 min, respectively, whereas participants from Minneapolis and Los Angeles walked less for transportation with median values of 120 min and 105 min, respectively.

### Built environment exposures in Euclidean buffers and network buffers

Table 2 shows the descriptive statistics for the counts of walkable destinations and frequent social destinations measured in Euclidean buffers and Network buffers for MESA participants across the six sites. The count of walkable destinations in Euclidean buffers were larger than the count of walkable destinations in network buffers. For example, for walkable destinations, the median counts in the 0.25-km, 1-km, and 5-km Euclidean buffers were 14, 233, and 4600, respectively, whereas the median counts of walkable destinations in the 0.25-km, 1-km, 5-km network buffers were 5, 125, and 2961, respectively. The relative difference in the counts comparing those in the Euclidian buffer to the network buffer was smaller in the larger buffers. The median count of walkable destinations in the 1-km Euclidian buffer was 1.8 times larger than the median count in the network buffer of the same size; the same relative comparison was 1.5 for the 5-km buffers, and 2.8 for the 0.25-km buffers. The counts of frequent social destinations in Euclidean and network buffers showed similar patterns.

**Table 2:** Descriptive statistics for walkable destinations and frequent social destinations for MESA participants in 2000 (N = 5756)

Walkable destinations	0.25-km		1-km		5-km	
	Euclidean buffers	Network buffers	Euclidean buffers	Network buffers	Euclidean buffers	Network buffers
	Median (Q1–Q3)	Median (Q1–Q3)	Median (Q1–Q3)	Median (Q1–Q3)	Median (Q1–Q3)	Median (Q1–Q3)
All sites	14 (3, 47)	5 (1, 17)	231 (103, 619)	123 (43, 334)	4583 (2575, 10,099)	2934 (1627, 7098)
NC (N = 891)	2 (1, 5)	1 (0, 2)	48 (15.5, 100)	15 (5, 42)	1303 (719, 2167)	668 (310, 1370.5)
NY (N = 921)	103 (61, 157)	26 (10, 46)	1173 (906, 1564)	611 (418, 808)	18,038 (15,343, 25,198)	12,623 (10,154, 17,645)
MD (N = 864)	7 (3, 23)	2 (0, 7)	163.5 (80, 303.25)	68 (28, 168)	3350 (2138.5, 6609.25)	2070.5 (1359, 3968.75)
MN (N = 945)	9 (4, 21)	5 (2, 11)	160 (102, 226)	94 (54, 143)	3353 (2773, 4465)	2249 (1740, 3019)
IL (N = 995)	36 (13, 87)	12 (2, 28)	491 (285.5, 1182.5)	259 (119, 641.5)	6865 (4441, 12,163)	4432 (2876.5, 9692)
CA (N = 1140)	11 (4, 29)	4 (1, 12)	246.5 (149.75, 387)	132 (64, 228)	5002 (3832, 7094.5)	3298 (2357.75, 4662.25)

Frequent social destinations	0.25-km		1-km		5-km	
	Euclidean buffers	Network buffers	Euclidean buffers	Network buffers	Euclidean buffers	Network buffers
	Median (Q1–Q3)	Median (Q1–Q3)	Median (Q1–Q3)	Median (Q1–Q3)	Median (Q1–Q3)	Median (Q1–Q3)
All sites	4 (1, 15)	1 (0, 5)	77 (34, 201)	41 (14, 106)	1487 (797, 2935)	969 (488, 2132)
NC (N = 891)	1 (0, 2)	0 (0, 1)	18 (7, 39)	6 (2, 17)	503 (266.5, 776.5)	245 (133, 475)
NY (N = 921)	29 (16, 45)	6 (2, 13)	332 (259, 470)	174 (119, 233)	5436 (4702, 6928)	3829 (3120, 4900)
MD (N = 864)	3 (1, 9)	1 (0, 3)	58 (26, 130.25)	25.5 (9, 68)	1313 (718.75, 2348.5)	780.5 (455, 1547.25)
MN (N = 945)	3 (1, 7)	1 (0, 4)	48 (32, 65)	28 (16, 42)	859 (715, 1180)	550 (422, 771)
IL (N = 995)	12 (3, 25.5)	4 (1, 9)	162 (89, 337.5)	79 (38.5, 180)	2204 (1670, 2861)	1522 (1043, 2160)
CA (N = 1140)	4 (1, 10)	1 (0, 4)	83 (45, 134)	43 (20, 80)	1589 (1180, 2401.5)	1061.5 (735.75, 1599)

*NC* North Carolina, *NY* New York, *MD* Maryland, *MN* Minnesota, *IL* Illinois, *CA* California

As expected, there were differences in the number of destinations across the six sites. The New York site had the highest number of walkable destinations and frequent social destinations, whereas the North Carolina site had the smallest number of walkable destinations and frequent social destinations. In addition, the number of walkable destinations in Euclidean buffers was around 1.5–3 times larger than the number of walkable destinations in network buffers depending on the site as well as buffer size. The number of frequent social destinations in Euclidean buffers relative to network buffers followed these patterns.

### Simulation results

We discuss the simulation results by comparing the difference in model evaluation metrics in the case where the model uses Euclidean buffer-based data, as opposed to the true network buffer-based data, across site, buffer size, built environment category and effect size.

**Fig. 1:**
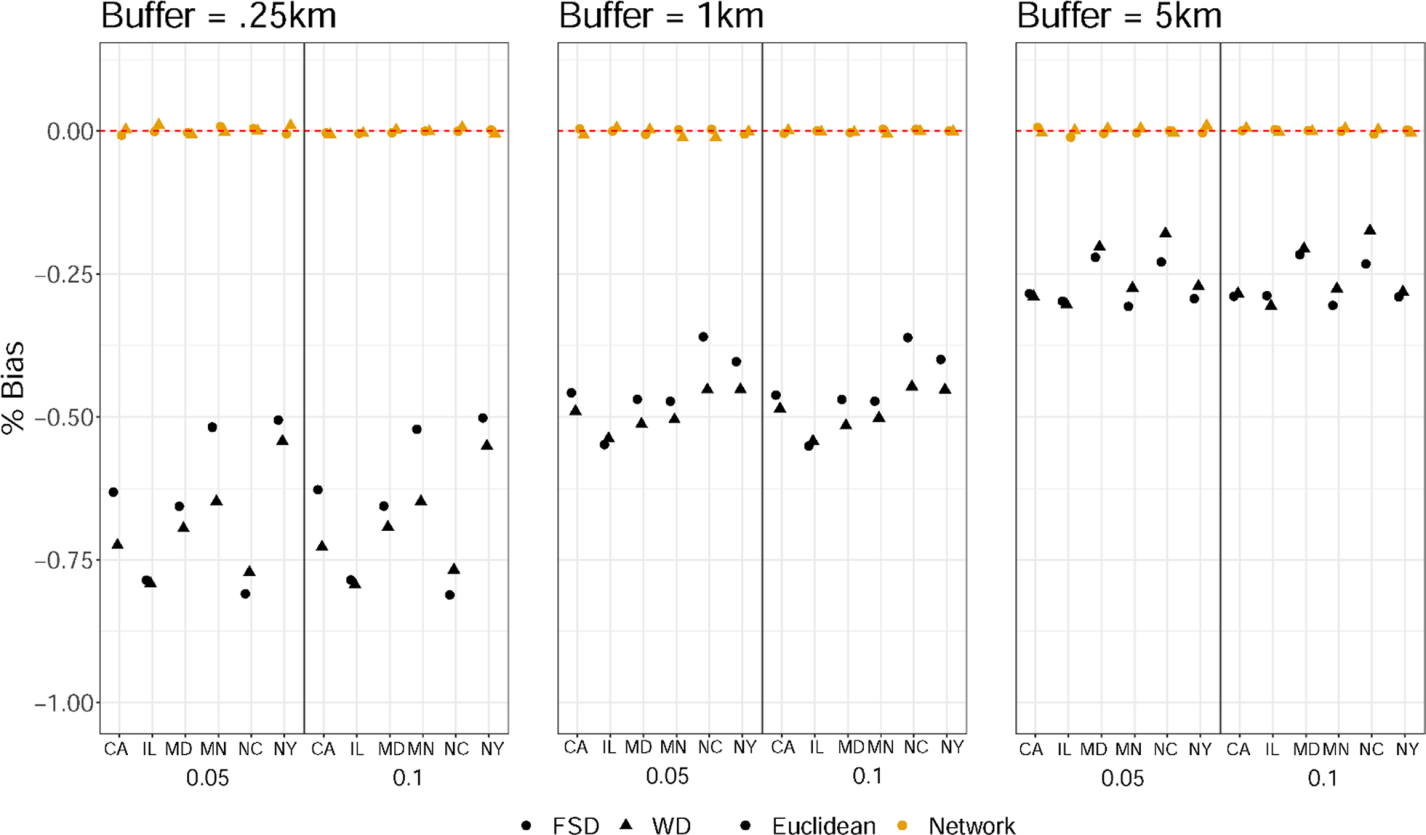
Simulation results for the degree of bias in associations between built environment exposures and transport walking when exposures were operationalized using 0.25 km, 1 km, and 5 km Euclidean buffers rather than network buffers, assuming network distances more accurately captured true exposures. FSD denotes frequent social destinations, WD denotes walkable destinations. 0.05 and 0.1 at the X-axis indicate a smaller (~ 0.05) and a larger (~ 0.1) built environment effect β. NC denotes North Carolina, NY denotes New York, MD denotes Maryland, MN denotes Minnesota, IL denotes Illinois, CA denotes California

As shown in Fig. 1, the bias in regression coefficients induced by using the Euclidean buffer-based counts was consistently negative, but there was little to no bias when the network based counts were used as the exposure measure. When using the Euclidean buffer-based counts, the bias in regression coefficients varied across buffer size and site, but to a smaller degree by built environment category or true effects size. For example, bias ranged from approximately − 80% in the North Carolina site for the FSD counts within 0.25 km, − 60% in the IL site for the FSD counts within 1 km, to roughly -20% in the North Carolina site for the WD counts within 5 km. The magnitude of percent bias was uniformly smaller for the 5 km spatial scale than the 1 km and 0.25 km spatial scales, independent of site or built environment category. For the 5-km buffer size, the percent bias ranged from 20 to 30% depending on site, but ranged across sties from 40 to 60% for the 1 km buffer, and from 50 to 80% for the 0.25 km buffer. Little differences in bias were observed across built environment categories or true effect sizes, within a given site.

**Fig. 2:**
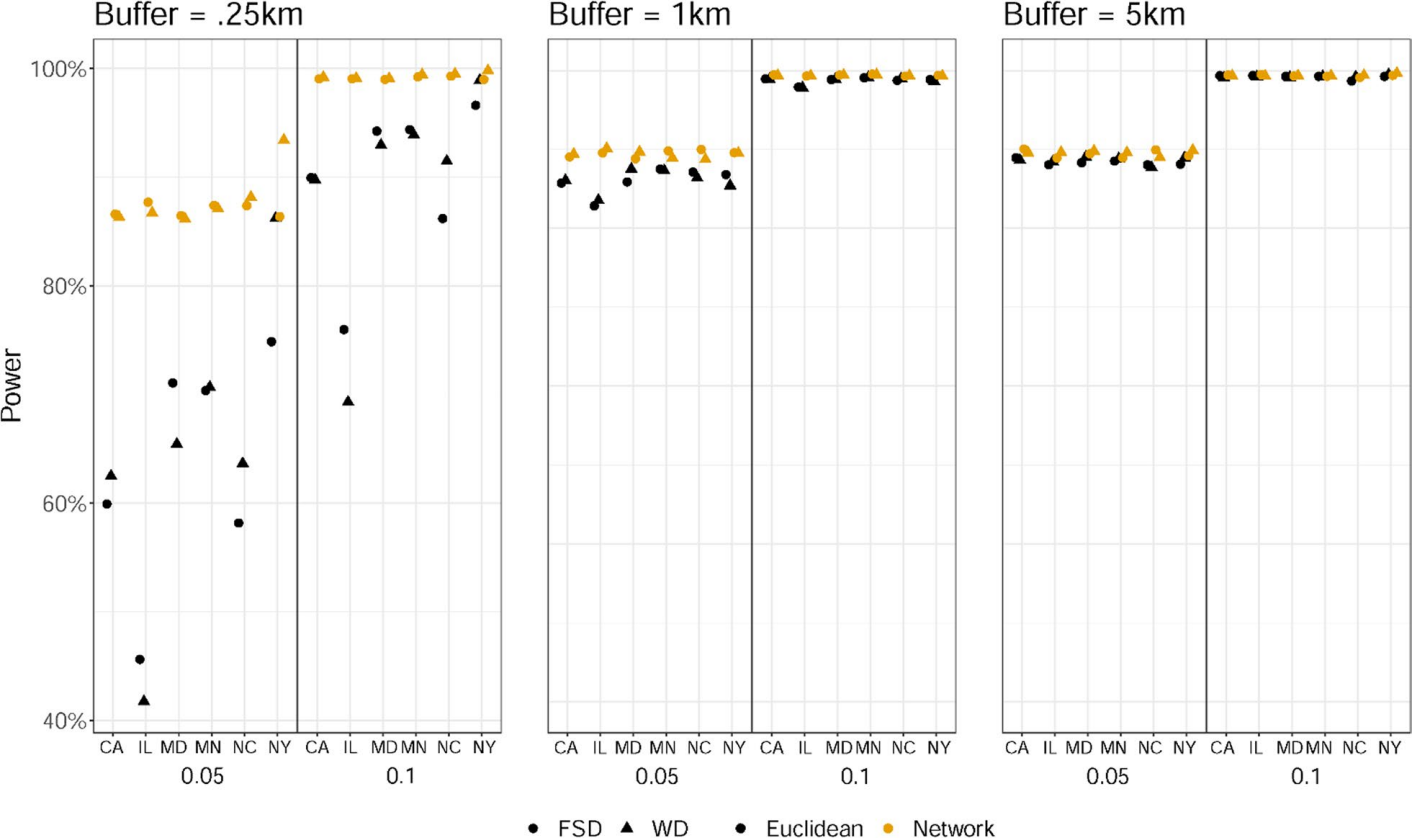
The simulation power in associations between built environment exposures and transport walking when exposures were operationalized using 0.25 km, 1 km, and 5 km Euclidean buffers rather than network buffers, assuming network distances more accurately captured true exposures. FSD denotes frequent social destinations, WD denotes walkable destinations. 0.05 and 0.1 at the X-axis indicate a smaller (~ 0.05) and a larger (~ 0.1) built environment effect β. NC denotes North Carolina, NY denotes New York, MD denotes Maryland, MN denotes Minnesota, IL denotes Illinois, CA denotes California

As shown in Fig. 2, power to detect associations when using the Euclidean buffer counts varied across different spatial scales. Compared to using network buffer counts, the power to detect associations using Euclidean buffer counts was much lower in the 0.25 km spatial scale regardless of effect size, built environment category, and site, whereas the power was only slightly lower or almost the same in the 1 km spatial scale or 5 km spatial scale. For example, for the 0.25 km spatial scale at the effect size of 0.05, the power using Euclidian buffer counts ranged from 40 to 10% lower than when using network buffers. For the 1 km spatial scale at the effect size of 0.05, using Euclidian buffer counts had < 10% lower power than when using network buffers. Differences in power were negligible for the 1 km spatial scale at effect size of 0.1 and for the 5 km spatial scale at both effect sizes. Although estimates obtained when using the Euclidian buffer counts were biased toward the null, power to detect associations did not deteriorate because the length of the confidence intervals for the Euclidean-based estimates tended to be narrower. Finally, power was uniformly stronger for the larger 5 km spatial scale than the smaller 1 km and 0.25 km spatial scales regardless of different sites, different built environment effect sizes, different built environment categories, and different buffer metrics used.

### Comparison of empirical network and Euclidean buffer-based associations

Table 3 shows the empirical associations between transport walking and built environment destinations in Euclidean buffers and network buffers for MESA participants. We found that the associations in network buffers were stronger than the associations in Euclidean buffers with the same spatial scale. For example, transport walking minutes increased by 3.94% with ten additional walkable destinations in the 0.25-km network buffer, whereas transport walking minutes increased by 1.35% with ten additional walkable destinations in the 0.25-km Euclidean buffer. Similarly, in the 0.25-km spatial scale, transport walking minutes increased by 9.99% with ten additional frequent social destinations in the network buffer, whereas transport walking minutes increased by 2.55% with ten additional frequent social destinations in the Euclidean buffer.

**Table 3:** Empirical associations between transport walking and built environment categories in network buffers and Euclidean buffers with spatial scales of 0.25-km, 1-km, 5-km, respectively (N = 5756)

Built environment categories	Percent change in transport walking minutes per week by 10 additional built environment destinations					
	Network buffer			Euclidean buffer		
	Coef	95% C.I		Coef	95% C.I	
Walkable destinations						
0.25-km	**3.94%**	1.15%	6.81%	**1.35%**	0.35%	2.36%
1-km	**0.30%**	0.08%	0.53%	**0.21%**	0.08%	0.33%
5-km	**0.02%**	0.01%	0.04%	**0.02%**	0.00%	0.03%
Frequent social destinations						
0.25-km	**9.99%**	1.00%	19.78%	2.55%	-0.45%	5.65%
1-km	0.67%	− 0.11%	1.45%	**0.61%**	0.14%	1.08%
5-km	**0.07%**	0.01%	0.13%	**0.05%**	0.00%	0.09%

Each model adjusts covariates: age, gender, race, education, income wealth index, BMI, self-rated health, arthritis last 2 weeks, car ownership, marital status, employment status, population density in 1-mile buffer around residence, street NetRatio in 1-mile buffer around residence. Bold fonts indicate p < 0.05

## Discussion

This study aimed to systematically quantify differences in the estimated associations between built environment exposures and health behaviors such as transport walking when exposure metrics were measured in Euclidean buffers and network buffers, separately. First, we used a simulation study to examine this question. Second, we examined the same question using empirical data from MESA study.

The simulation study showed that the bias induced by using exposure counts derived from Euclidean buffers was consistently negative, irrespective of spatial scale, context, effect size or exposure category. Assuming that network buffers more closely approximate the causally relevant geographic context [14, 22], the simulation results suggest that compared with network buffers, built environment exposures measured using Euclidean buffers underestimate exposure effects on transport walking. A potential reason is that the size of a Euclidean buffer is larger than the size of a network buffer with the same radius [29], and thus encompasses more built environment destinations some of which may not be relevant to the outcome assessed. Thus, the average effects of all the included built environment destinations in the Euclidean buffers are ultimately attenuated. In other words, downward bias in the Euclidian-based estimates is directly related the percent of built environment features incorrectly included in a Euclidian buffer. This finding is in line with results in previous literature that health impacts of built environment features in Euclidean buffers were weaker than those in network buffers [22]. The empirical results using MESA data showed a consistent pattern with the simulation results. Whereas prior studies have used one dataset at a time or one geographic context, our simulation study demonstrated that attenuation of effects when using Euclidian buffers occur across a variety of spatial scales, contexts, effect sizes, and density of built environment features.

The simulation study also showed that the bias induced by using exposure counts derived from Euclidean buffers exhibited variation, ranging from approximately − 80% to roughly − 20% across the scenarios examined. This variation in bias was primarily driven by the spatial scale used. When a larger spatial scale was used, the percent of bias tended to be smaller. This is likely because the percent of built environment features incorrectly included in Euclidian buffers tends to be smaller in larger buffers compared to the smaller buffers. Thus, using Euclidian buffers counts paired with smaller spatial scales will likely result in a larger percent of incorrectly included built environment features in exposure count and thus larger bias. The choice of using network-based vs. Euclidian-based exposures is more important when the relevant spatial scale is likely to be smaller.

There was also some variation in the magnitude of the bias in the Euclidian-based associations across contexts (the six MESA sites), and little variation by built environment categories (FSD/WD). Independent of the context, using the Euclidian buffers always leads to incorrect inclusion of some BEFs in the exposure count. However, the percent of incorrectly included built environment features (and thus bias) was larger between contexts than between categories within contexts. Differences in overcount across contexts may be due to differences in the street-network layout of different contexts, and thus lead to larger/smaller degree of overcount across contexts. However, within a context, the relative overcount of destinations within the Euclidian buffer vs. the network buffer is similar across the categories. This makes sense, since the network layout supersedes the placement of a particular type of destination (FSD or WD), thus context matters more than BEF category.

Ultimately, biased associations will be present when the spatial shape used to assess exposure does not match true causally relevant geographic context, providing an example of the Uncertain Geographic Context Problem [17]. In this paper, we showed that when the spatial shape used Euclidian buffer results in a systematic overcount of exposure count compared to the truth (here assumed to be count in the network buffer), then the association estimated will have bias toward the null. When a person’s activity space is smaller than the spatial unit used for exposure assessment, such as when county or other large administrative unit is used, then associations will have negative bias.

## Strengths and limitations

This study is the first to quantify the degree of bias when exposures were operationalized using Euclidean buffers rather than network buffers (assuming network buffers more accurately captured true exposures). Second, this study examined the associations between built environment and transport walking based on a multi-ethnic and multi-site data, which could help generate more generalizable knowledge across different population groups and various geographic settings. Third, this study examined the extent of bias across multiple sites, highlighting that context plays an important role in the magnitude of the bias. Further, this study quantified the extent of bias across different spatial scales and different built environment categories (or densities), informing future research how the bias shifts by spatial scale and by built environment category (or density).

There are also some limitations in this study. First, the transport walking minutes per week for the MESA participants were self-reported. Future research could focus on the transport walking minutes based on pedometer or accelerometers. Second, while we focused on built environment exposures within buffers around residence, we did not account for built environment exposures in activity spaces along the course of daily activities. GPS tracking data provide a promising fashion for exploring the health impacts of built environments in activity spaces. Third, the participants in our study sample were mostly middle-age and older adults, thus, our empirical findings might not be generalizable to all adults or children. Further, we did not examine to what degree exposure metrics may be influenced by incomplete road network data since MESA participants reside in urbanized areas with well documented/largely complete street network data. However, future studies may examine the impact of the uncertainty by comparing different road network datasets with different levels of completeness.

## Conclusions

This study contributes to the literature in several ways. First, this study is the first to quantify the extent of bias in the magnitude of the associations between built environment exposures and transport walking when the former are measured in Euclidean buffers vs. network buffers. Simulation results found that the bias induced by using Euclidean buffer models was consistently negative across the six study sites, suggesting built environment exposures measured using Euclidean buffers underestimate associations with transport walking. This finding helps inform future research to carefully conceptualize appropriate buffer metrics to characterize built environment features. Second, the extent of bias in the associations between built environment exposures measured in Euclidean buffers and transport walking helps contextualize existing research in the field that uses Euclidean buffers when that are the only option. Further, this study provides an example of the Uncertain Geographic Context Problem, highlighting that built environment exposures and their associations with transport walking are sensitive to different spatial contexts with varying shapes and sizes.

## Supplementary Information

**Additional file 1: Table S1**. List of walkable destinations and a subdomain used in this study

## Data Availability

The data that support the findings of this study are available from Multi-Ethnic Study of Atherosclerosis (MESA), but restrictions apply to the availability of these data, which were used under license for the current study, and so are not publicly available.
