# Supplementary material for: Comparing effects of Euclidean buffers and network buffers on associations between built environment and transport walking: the Multi-Ethnic Study of Atherosclerosis
Source: Int J Health Geogr. 2022 Sep 17;21:12. doi: 10.1186/s12942-022-00310-7 (PMC9482303; doi:10.1186/s12942-022-00310-7)
Supplement: Supplementary file 1 — Additional file 1: Table S1. List of walkable destinations and a subdomain used in this study [file 12942_2022_310_MOESM1_ESM.docx]

**Supplement Materials**

Supplement Table 1. List of walkable destinations and a subdomain used in this study

| **Primary Domain** | **Destinations** |
| --- | --- |
| 1. Walkable destinations | Small grocers/bodegas/medium-sized grocers |
|  | Bakery, Candy, Ice Cream, Coffee shops |
|  | Convenience stores |
|  | Restaurants/Eating places/Fast food |
|  | Mass Merchandiser/Wholesale/warehouse/Supercenters/Supermarkets |
|  | Other food stores/Department Stores |
|  | Bars and nightclubs serving alcohol/Liquor stores |
|  | Beauty shops and barbers |
|  | Banks and Credit unions |
|  | Pharmacies/Drug Stores |
|  | Laundromat/Dry cleaning |
|  | Libraries |
|  | U.S. Postal Service |
|  | Light/moderate/vigorous/multi-use physical activity |
|  | Massage parlors/Spas/Tanning Salons |
|  | Amusement parks, carnival, rodeo/arcades/gambling |
|  | Day care/Preschool/Elementary and Secondary schools/Colleges/Universities |
|  | Museum and art gallery/Zoo, aquarium, arboretum |
|  | Performance based entertainment/Sports and stadium entertainment |
|  | Political Organizations/Religious institutions |
|  | Social clubs/Recreation Clubs/Individual and family social services |
| **Sub-domains** | **Destinations** |
| 1.1. Frequent social destinations | Light/moderate/vigorous/multi-use physical activity |
|  | Beauty shop/Barber |
|  | Libraries |
|  | Non-physical activity recreation clubs/ Religion institutions |
